# Dehydroandrographolide Alleviates Oxidative Stress, Inflammatory Response, and Pyroptosis in DSS-Induced Colitis Mice by Modulating Nrf2 Signaling Pathway

**DOI:** 10.3390/biom15111580

**Published:** 2025-11-10

**Authors:** Meifen Wang, Zhenyu Li, Xinghua Lei, Ziyue Yang, Shuixing Yu, Guangxin Chen

**Affiliations:** 1 Institutes of Biomedical Sciences, Shanxi University, Taiyuan 030006, China; wangmeifen@sxu.edu.cn (M.W.); leixinghua@sxu.edu.cn (X.L.); yangziyue64@outlook.com (Z.Y.); 2 State Key Laboratory of Reproductive Regulation and Breeding of Grassland Livestock, College of Life Sciences, Inner Mongolia University, Hohhot 010070, China; 15534230622@163.com; 3 Shanxi Provincial Key Laboratory of Medical Molecular Cell Biology, Taiyuan 030006, China

**Keywords:** inflammatory bowel disease, DA, inflammatory response, oxidative stress, pyroptosis

## Abstract

Dehydroandrographolide (DA), a bioactive diterpenoid from *Andrographis paniculata* with diverse biological activity, was investigated for its antioxidant and anti-inflammatory effects in lipopolysaccharide (LPS)-stimulated RAW264.7 macrophages and dextran sulfate sodium (DSS)-induced murine colitis. In vitro, DA inhibited the inflammatory response by modulating extracellular Signal-Regulated Kinase (Erk), c-Jun N-terminal Kinase (Jnk), p38 Mitogen-Activated Protein Kinase (P38), nuclear factor kappa-light-chain-enhancer of activated B cells (NF-κB) p65 activation, and downregulated *interleukin-6* (*il-6*) and *interleukin-1β* (*il-1β*) mRNA. It also had antioxidant effects by upregulating Nuclear Factor Erythroid 2-Related Factor 2 (Nrf2), NAD(P)H quinone dehydrogenase 1 (Nqo-1) and heme oxygenase-1 (Ho-1), promoting protein kinase B (Akt) and 5′-adenosine monophosphate-activated protein kinase-α1 (Ampk-α1) phosphorylation. DA decreased cyclooxygenase-2 (Cox-2) and inducible nitric oxide synthase (iNos) levels and alleviated intracellular reactive oxygen species (ROS) accumulation. In vivo, DA alleviated DSS-induced colitis in wild type (WT) mice by improving weight loss, disease activity index, colonic inflammation, and oxidative stress. The beneficial effects were linked to inhibiting Erk, Jnk, and P38 activation and enhancing Nrf2 signaling pathway. DA inhibited NOD-like receptor family pyrin domain-containing 3 (Nlrp3) inflammasome-mediated pryoptosis. However, DA’s protective effects were abolished in DSS-induced *nrf2*^−/−^ mice, suggesting its efficacy depends on Nrf2 signaling. Overall, DA alleviates oxidative stress, inflammatory responses, and pyroptosis in experimental colitis mice mainly by activating Nrf2 signaling pathway, highlighting its potential as a promising therapeutic option for inflammatory bowel disease.

## 1. Introduction

The intestine serves not only as the primary site for nutrient digestion and absorption but also as a crucial barrier against external pathogens and toxins. Maintaining its homeostasis is of utmost importance. Its disruption, such as alterations in the gut microbiota composition or abnormalities in intestinal immune function, can trigger a variety of intestinal disorders. Among these, inflammatory bowel diseases (IBDs) are the most common chronic inflammatory conditions of the gastrointestinal tract, imposing an increasingly significant global public health burden. IBD represents a heterogeneous group of chronic inflammatory disorders, affecting approximately 0.3–0.5% of the global population [1], with notable regional variations in prevalence. Partial differential equations were developed to model time dependent prevalence for three stage 3 regions [2,3]: Canada, 0.65% in 2014 to 0.83% in 2025 to 0.96% in 2035 to 1.05% in 2043; Denmark, 0.86% in 2014 to 1.19% in 2025 to 1.44% in 2035 to 1.59% in 2043; and Scotland, 0.74% in 2014 to 1.04% in 2025 to 1.32% in 2035 to 1.51% in 2043 [3].

IBDs mainly include Ulcerative colitis (UC) and Crohn’s disease (CD) [4,5]. Historically regarded as a “Western disease”, IBD has become a global health issue since the 21st century. Its rising prevalence in developing countries threatens health and brings economic burdens. Although monoclonal antibody-based biologics have shown effectiveness in managing IBD [6], many patients ultimately lose responsiveness or fail to achieve remission [7], emphasizing the urgent need to develop novel therapeutic strategies [8]. Clinically, these disorders are characterized by a range of symptoms, such as mucosal ulceration, involuntary weight loss, chronic diarrhea, hematochezia [9], and abdominal cramping [10]. Pathologically, IBDs are hallmarked by a persistent breakdown of the colonic epithelial barrier, impaired epithelial integrity, and an excessive and dysregulated intestinal inflammatory response [11]. The manifestations of IBD display significant heterogeneity regarding disease severity and anatomical location within the gastrointestinal tract. Its pathogenesis remains incompletely understood; however, growing evidence implicates gut dysbiosis, genetic susceptibility, environmental triggers, and aberrant intestinal immune activation as key factors driving the onset and progression of the disease [12].

Dehydroandrographolide (DA), a major active component of the traditional oriental medicinal herb *Andrographis paniculata* [13,14], has been used for the treatment of infections [15] and inflammatory disorders [16]. It exhibits diverse pharmacological activities, including antibacterial, anti-inflammatory, and antiviral properties that are comparable to those of natural antiviral or anti-inflammatory drugs [17]. Previous research has shown DA’s hepatoprotective and antifibrotic properties [18], inhibition of oral cancer cell migration [19], and suppression of allergic reactions [20]. The anti-inflammatory efficacy of DA in various conditions, such as arthritis [21], lung injury [22], and live injury [23], suggesting its systemic anti-inflammatory potential. Nevertheless, its impacts on intestinal inflammatory injury, especially in non-infectious inflammatory bowel disorders remain unexplored. Considering the crucial role of macrophages in promoting intestinal inflammation, targeting macrophage-mediated inflammatory responses is a key therapeutic strategy [24]. Therefore, this study aimed to explore the antioxidant and anti-inflammatory effects of DA in LPS-stimulated macrophages and DSS-induced murine colitis model, providing a theoretical basis for the application of DA in the treatment of IBDs.

## 2. Materials and Methods

### 2.1. DA Reagent

DA is a bioactive diterpenoid isolated from *Andrographis paniculata*, was obtained from Yuanye Biotechnology (Shanghai, China), the product number is B25335, and the purity is ≥99.7%.

### 2.2. Animal Model

Adult male wild type (WT) and *nrf2*^−/−^ C57BL/6 mice, aged 6–8 weeks, were utilized, and housed at 22–23 °C under a 12 h light/dark cycle, with food and water ad libitum.

WT mice: The mice were assigned to three groups with 12 mice per group. The control group was maintained under standard conditions. In the DSS group, colitis was induced by administering 2.5% DSS in drinking water for 5 days. In the DSS + DA group, mice were administered DA (50 mg/kg) intragastrically daily for 7 days [23], followed by the administration of 2.5% DSS in drinking water for 5 days.

WT and *nrf2*^−/−^ mice: The mice were divided into six groups, with 11 mice in each group. The WT and *nrf2*^−/−^ groups were maintained under standard conditions. The WT + DSS and *nrf2*^−/−^ + DSS groups were provided with 2.5% DSS in drinking water for 7 days. The WT + DSS + DA and *nrf2*^−/−^ + DSS + DA groups were administered DA (50 mg/kg) intragastrically daily for 7 days, followed by the administration of 2.5% DSS in drinking water for 7 days. After the DSS treatment was completed, the mice were sacrificed, and the colon tissues were collected.

Both WT and *nrf2^−/−^* mice were purchased from Cyagen Biosciences Inc. (Suzhou, China). All experimental procedures were approved by the Institutional Animal Care and Use Committee of Shanxi University (SXULL2019004, 15 April 2019).

### 2.3. Disease Activity Index

During the establishment of DSS-induced colitis models in mice, body weight, stool consistency, and fecal occult blood were monitored. Disease activity index (DAI) scores were calculated based on established criteria [25], incorporating weight loss, stool characteristics, and bleeding severity.

### 2.4. Hematoxylin and Eosin (H&E) and Immunohistochemistry

Mouse colon tissues were rinsed in PBS, fixed with 4% paraformaldehyde, paraffin-embedded, sectioned at 5 μm, and stained with hematoxylin and eosin (H&E). Immunohistochemistry, the 5-μm paraffin sections of the colon were processed in accordance with the immunohistochemistry kit of Boster (SA1050, Wuhan, China). The primary antibody, Muc2, was sourced from ZEN-BIOSCIENCE (Cat#R381746, 1:200, Chengdu, China) and used at a dilution ratio of 1:300. The DAB chromogenic kit was also procured from Boster (AR1027, Wuhan, China). Following the completion of staining, the sections were mounted with neutral resin. Subsequently, the sections were examined and photographed under a microscope (ZEISS, Imager.M2, AOberkochen, Germany).

### 2.5. Cell Culture

RAW264.7 cells, a murine macrophage cell line, were obtained from BeNa Culture Collection (Beijing, China). These cells were cultured in Dulbecco’s modified Eagle’s medium (DMEM; Gibco, Grand Island, NY, USA) supplemented with 10% fetal bovine serum (FBS; Sorfa,, Beijing, China) and 1% penicillin and streptomycin (NCM Biotech, Suzhou China). The cells were maintained at 37 °C in a humidified incubator with 5% CO_2_.

### 2.6. Cell Viability Assay

RAW 264.7 cells (1.0 × 10^4^ cells per well) were seeded in 96-well plates and pre-treated with 1, 2, 10, 20, 60, and 100 μg/mL of DA for 24 h. Subsequently, 10 μL of CCK-8 solution (1 mg/mL) was added to each well, and the plates were incubated for 30 min. Finally, 10 μL of dimethyl sulfoxide (DMSO) (Solarbio, Beijing, China) was added to each well. The absorbance was measured at 450 nm using a microplate reader (Nikon, Tokyo, Japan).

### 2.7. Western Blot

Total protein was isolated from RAW264.7 cells and mouse colon tissue using ice-cold radioimmunoprecipitation assay (RIPA) lysis buffer (Solarbio, Beijing, China) containing 1% phenylmethylsulfonyl fluoride (PMSF) and phosphatase inhibitors (Solarbio, Beijing, China). The homogenates were incubated at 4 °C for 30 min, followed by centrifugation at 12,000× *g* for 10 min at 4 °C. The resulting supernatant was then collected for analysis. Protein concentration was measured using a Pierce BCA Protein Assay Kit (Thermo Fisher Scientific, Waltham, MA, USA). Equal amounts of protein from each sample were separated by 10% SDS-PAGE and electrophoretically transferred to 0.45 µm PVDF membranes. The membranes were blocked with 5% non-fat milk in TBST for 1 h at room temperature, then incubated with specific primary antibodies at 4 °C overnight. The primary antibodies used in this study were as follows: anti-Nrf2 (Bioswamp, Cat#PAB30175, 1:2000, Shanghai, China), anti-Ho-1 (Bioswamp, Cat#PAB38338, 1:2000, China), anti-Nqo-1 (Bioswamp, Cat#PAB32354, 1:2000, shanghai, China), and anti-β-actin (Bioswamp, Cat#MAB48206, 1:10000, Shanghai, China); anti-Ampk-α1 (ABclonal, Cat#A27740, 1:2000, Wuhan, China), anti-phospho-Ampk-α1 (ABclonal, Cat#AP0871, 1:2000, Wuhan, China), anti-Akt (ABclonal, Cat#AF6261, 1:2000, Wuhan, China), anti-phospho-Akt (ABclonal, Cat#AF0016, 1:2000, Wuhan, China), and anti-Gapdh (ABclonal, Cat#AC001, 1:10,000, Wuhan, China); anti-Erk (ZEN-BIOSCIENCE, Cat#343830, 1:1000, Nanjing, China), anti-phospho-Erk (ZEN-BIOSCIENCE, Cat#301245, 1:1000, Nanjing, China), anti-Jnk (ZEN-BIOSCIENCE, Cat#R24780, 1:1000, China), anti-phospho-Jnk (ZEN-BIOSCIENCE, Cat#R381100, 1:1000, China), anti-P38 (ZEN-BIOSCIENCE, Cat#200782, 1:1000, China), and anti-phospho-P38 (ZEN-BIOSCIENCE, Cat#310091, 1:1000, Nanjing, China); anti-Nlrp3(abcam, Cat#ab91413, 1:1000, Cambridge, UK), anti-Caspase-1 (Immunoway, Cat#YM8437, 1:2000, Beijing, China), and anti-Gsdmd-NT (Immunoway, Cat#YT7991, 1:2000, Beijing, China). After the primary incubation, the membranes were washed with TBST and then incubated with goat anti-rabbit or goat anti-mouse secondary antibodies (ABclonal; diluted 1:10,000, Wuhan, China) for 1 h at room temperature. Following thorough washing, the protein bands were visualized using an enhanced chemiluminescence (ECL) kit (Applygen Technologies, Beijing, China) and detected with an Amersham Imager 600 system (GE Healthcare, Chicago, IL, USA). The intensity of the bands was quantified using ImageJ software (NIH, Bethesda, MD, USA, version 1.4.3.67) and normalized to the Gapdh or β-actin signal.

### 2.8. Real-Time Quantitative PCR

Total RNA from cells was isolated using Trizol reagent (TransGen Biotech, Beijing, China) following the manufacturer’s instructions. The RNA samples were treated with DNase I (Sigma, St. Louis, MI, USA), quantified, and reverse transcribed into cDNA using the TransScript first-strand cDNA synthesis SuperMix (TransGen Biotech, Beijing, China). Real-time quantitative PCR was carried out using SYBR^®^ Premix Ex Taq™ II (Tli RNase H Plus) (TaKaRa, Dalian, China) in the ABI PRISM^®^ 7500 real-time PCR system (Applied Biosystems, Foster City, CA, USA). The primer sequences employed in this study were obtained from the PrimerBank website. The specificity of PCR was verified by analyzing the melting curves, and the data were analyzed using the comparative threshold cycle method. The primer sequences are presented below: *il-1β*, 5′-GTTCCCATTAGACAACTGCACTACAG-3′, 5′-GTCGTTGCTTGGTTCTCCTTGTA-3′; *il-6*, 5′-CCAGAAACCGCTATGAAGTTCC-3′, 5′-GTTGGGAGTGGTATCCTCTGTGA-3′; *tnf-α*, 5′-CCCCAAAGGGATGAGAAGTTC-3′, 5′-CCTCCACTTGGTGGTTTGCT-3′; *β-actin*, 5′-GTCAGGTCATCACTATCGGCAAT-3′, 5′-AGAGGTCTTTACGGATGTCAACGT-3′; *gapdh,* 5′-GGGGTCGTTGATGGCAACA-3′, 5′-AGGTCGGTGTGAACGGATTTG-3′.

### 2.9. Reactive Oxygen Species (ROS) Detection

RAW264.7 macrophages were seeded in 96-well plates and pre-incubated with 10 μg/mL DA for 1 h, then stimulated with LPS for 12 h. Intracellular ROS levels were measured using the Reactive Oxygen Species Assay Kit (Solarbio, Beijing, China), with 2′,7′-dichlorodihydrofluorescein diacetate (DCFH-DA) serving as the fluorescent probe. The procedure was strictly carried out in accordance with the manufacturer’s instructions. Fluorescence intensity was observed and captured under a fluorescence microscope (Nikon, Ti2-U, Tokyo, Japan).

### 2.10. MDA, GSH, and LDH Detection

- Malondialdehyde (MDA) Assay

MDA levels were measured using a commercial kit (BC0025, Solarbio, Beijing, China) according to the manufacturer’s protocol. Briefly, approximately 0.1 g of colon tissue was homogenized in 1 mL of tissue extraction solution using a cell and tissue homogenizer. The homogenate was centrifuged at 8000× *g* for 10 min at 4 °C, and the resulting supernatant (sample solution) was collected. For the reaction mixture, 300 μL of MDA detection working solution, 100 μL of the sample solution, and 100 μL of Reagent 3 were mixed. Distilled water replaced the sample solution as the blank control. The mixture was incubated at 100 °C for 1 h in a metal bath, cooled on ice, and centrifuged at 10,000× *g* for 10 min at room temperature. A 200 μL of the resulting supernatant was transferred to a 96-well plate, and absorbance was measured at 532 nm and 600 nm using a microplate reader (BioTek, H1M, Winooski, VT, USA). The MDA content was calculated using the formula provided in the kit instructions.

- Glutathione (GSH) Assay

The GSH concentration was measured using a commercial kit (BC1175, Solarbio, Beijing, China). Approximately 0.1 g of colon tissue was homogenized in 1 mL of Reagent 1 using a cell and tissue homogenizer. The homogenate was centrifuged at 12,000× *g* for 10 min at 4 °C, and the resulting supernatant (sample solution) was collected. For the reaction mixture, 20 μL of sample solution, 140 μL of Reagent 2, and 40 μL of Reagent 3 were mixed. Distilled water replaced the sample solution as the blank control. After standing at room temperature for 2 min, the absorbance was measured at 412 nm using a microplate reader. The GSH content was calculated based on the standard sample parameters provided in the kit.

- Lactate Dehydrogenase (LDH) Assay

LDH activity was measured using a commercial kit (BC0685, Solarbio, Beijing, China). Approximately 0.1 g of colon tissue was homogenized in 1 mL of Reagent 1 using a cell and tissue homogenizer. The homogenate was then centrifuged at 8000× *g* for 10 min at 4 °C, and the supernatant (sample solution) was collected. For the reaction mixture, 10 μL of the sample solution, 50 μL of Reagent 1, 10 μL of Reagent 2, 50 μL of Reagent 3, and 150 μL of Reagent 4 were mixed. In the blank control, distilled water was used to replace Reagent 2. After incubation at room temperature for 3 min, the absorbance was measured at 450 nm using a microplate reader. The LDH activity was calculated according to the standard sample parameters provided by the kit.

### 2.11. ELISA Analysis

The levels of Il-1β (ELK1271, ELK Biotechnology, Wuhan, China) and Il-18 (ELK2269, ELK Biotechnology, Wuhan, China) in colon tissues were measured using ELISA kits, following the manufacturer’s instructions. For sample preparation, colon tissues were homogenized, and total protein was extracted. Regarding standards and samples, 100 μL of standards (varying concentrations) or tissue lysates were added to a 96-well plate and incubated at 37 °C for 80 min. After incubation, the wells were washed 3 times with 200 μL of washing buffer and patted dry. Antibody incubation, 100 μL of biotinylated antibody was added and incubated at 37 °C for 50 min, followed by another 3 times washes. For HRP conjugation, 100 μL HRP enzyme solution was added and incubated at 37 °C for 50 min, followed by a thorough 5 times washes. For detection, 90 μL of TMB substrate was added and incubated at 37 °C for 20 min, and then the reaction was stopped with 50 μL stop solution. The absorbance was measured at 450 nm, and the concentrations were calculated.

### 2.12. Statistical Analysis

Data are presented as mean ± standard error of the mean (SEM). All statistical analyses were carried out using GraphPad Prism software (version 7.00, San Diego, CA, USA). One-way analysis of variance (ANOVA) was used for the comparison among multiple groups, followed by Tukey test for the comparison between groups. Statistical significance was defined as * *p* < 0.05, ** *p* < 0.01, *** *p* < 0.001, and **** *p* < 0.0001. Each experimental group consisted of at least three independent replicates.

## 3. Results

### 3.1. DA Inhibits Inflammatory Response in LPS-Induced Macrophages

**Figure 1:**
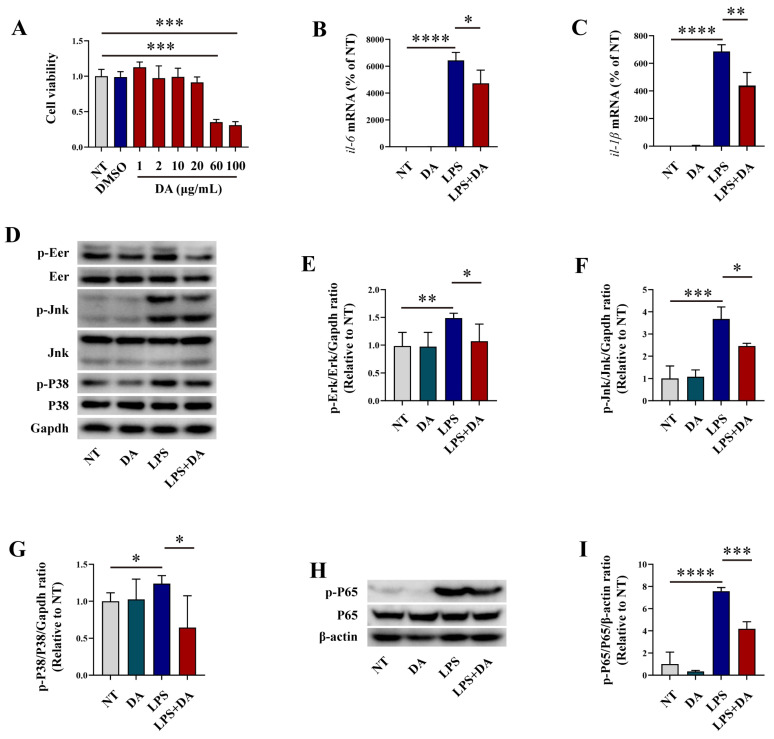
DA inhibits LPS-induced inflammatory response in macrophages. (**A**) The CCK8 assay was used to evaluate the effects of DA on macrophages viability. (**B**,**C**) qRT-PCR was employed to analyze the mRNA expression levels of *il-6* and *il-1β*. (**D**–**G**) WB analysis was performed to analyze the phosphorylation of Erk (**D**,**E**), Jnk (**D**,**F**), and P38 (**D**,**G**) signaling pathway in LPS-induced macrophages (**H**,**I**) WB was used to analyze the phosphorylation of the NF-κB p65 signaling pathway in LPS-induced macrophages. Data are presented as means ± SEM, * *p* < 0.05, ** *p* < 0.01, *** *p* < 0.001, and **** *p* < 0.0001.

Cell viability assays showed that DA at concentrations of 1, 2, 10, and 20 μg/mL had no impact on cell viability (Figure 1A). Based on these findings, 10 μg/mL of DA was chosen to explore its effect on the inflammatory response in LPS-induced macrophages. qRT-PCR analysis showed that DA had no effects on the mRNA expression of *il-6* and *il-1β*, and LPS upregulated their expression. However, pre-treatment with DA significantly reduced the expression of these pro-inflammatory mediators (Figure 1B,C). Mitogen-activated protein kinases (Mapks) pathways, which are classical mediators of inflammation activated by various stimuli to regulate inflammatory responses [26], represent potential targets for novel anti-inflammatory drugs [27]. Our results demonstrated that only DA had no effects on the phosphorylation of Erk, Jnk, and P38 Mapks, and LPS significantly increased their phosphorylation, while pre-treatment with DA markedly decreased Mapk phosphorylation (Figure 1D–G). Moreover, DA significantly inhibited the phosphorylation of NF-κB p65 in LPS-induced RAW264.7 macrophages (Figure 1H,I).

### 3.2. DA Inhibits Oxidative Stress in LPS-Induced Macrophages

**Figure 2:**
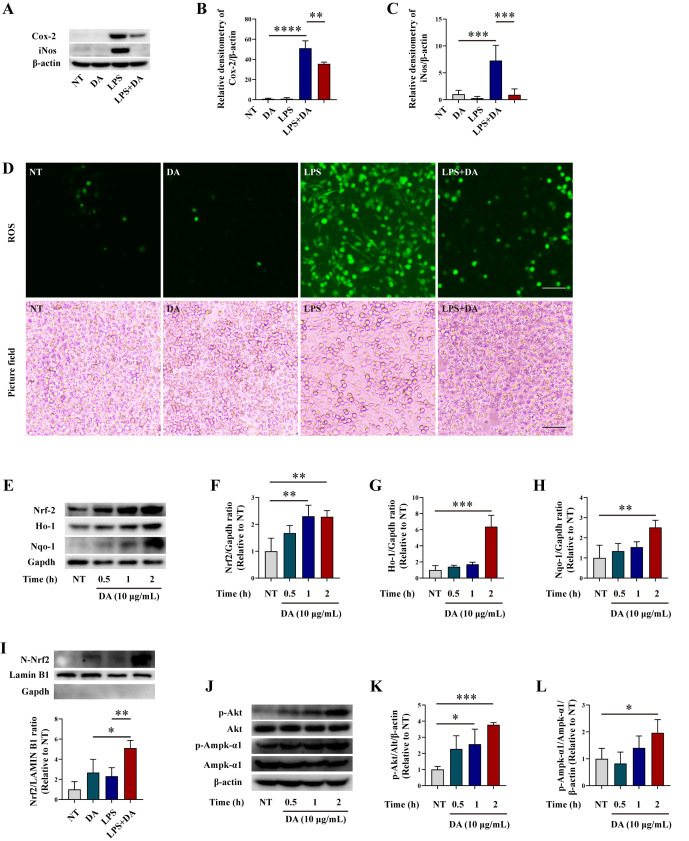
DA inhibits LPS-induced oxidative stress in macrophages. (**A**–**C**) WB analysis was performed to detect the protein levels of Cox-2 (**A**,**B**) and iNos (**A**,**C**) in LPS-induced macrophages. (**D**) The production of ROS was measured using a ROS assay kit and observed under a fluorescence microscope. Green representative ROS, the images were captured at 20× magnification, and the scale bar indicates 500 μm. (**E**–**H**) WB analysis was used to determine the protein levels of Nrf2 (**E**,**F**) Ho-1 (**E**,**G**), and Nqo-1 (**E**,**H**) in LPS-induced macrophages. (**I**) WB assay was employed to assess the effects of DA and LPS on the nuclear translocation of Nrf2. (**J**–**L**) WB assay was employed to assess the phosphorylation levels of Akt (**I**,**J**), and Ampk-α1 (**I**,**K**) in LPS-induced macrophages. Data are presented as means ± standard error of the mean (SEM). * *p* < 0.05, ** *p* < 0.01, *** *p* < 0.001, and **** *p* < 0.0001.

Subsequently, we investigated the effects of DA on oxidative stress in LPS-induced macrophages. Western blot analysis revealed that LPS significantly upregulated the expression of pro-inflammatory enzymes, including inducible nitric oxide synthase (iNos) and cyclooxygenase-2 (Cox-2). In contrast, pretreatment with DA significantly mitigated this effect (Figure 2A–C). Additionally, we observed that LPS treatment increased the level of ROS, which was effectively inhibited by pre-treatment with DA in macrophages (Figure 2D). Our findings demonstrated that pre-treatment with DA mitigates the inflammatory response and oxidative stress in LPS-induced macrophages. Nrf2 is the most critical transcription factor in counteracting oxidative stress and plays a key role in inhibiting oxidative stress and inflammatory response [28]. Consequently, in the subsequent step, we investigated the effects of DA on Nrf2, as well as its downstream targets Ho-1 and Nqo-1. Our findings revealed that treatment with DA significantly enhanced the expression of Nrf2, Ho-1, and Nqo-1 in macrophages (Figure 2E–H), suggesting that DA might exert anti-oxidative effects. Furthermore, both DA and LPS can promote the nuclear translocation of Nrf2 to varying degrees. Among them, the combination of DA and LPS exhibits the greatest capacity to promote the nuclear translocation of Nrf2 (Figure 2I), suggesting that DA can indeed facilitate the activation of Nrf2. Additionally, we evaluated the phosphorylation status of AMP-activated protein kinase α 1 (Ampk-α1) and Akt, which are known to facilitate Nrf2 stabilization and nuclear translocation. Western blot results demonstrated that treatment with DA promoted the phosphorylation level of the Ampk-1α/Akt signaling pathway (Figure 2J–L). Collectively, these data suggest that the antioxidant effects of DA may be associated with the phosphorylation of Ampk-α1/Akt, which subsequently enhances the activation of the Nrf2 signaling pathway.

### 3.3. DA Mitigates Intestinal Inflammatory Injury in DSS-Induced Mice

**Figure 3:**
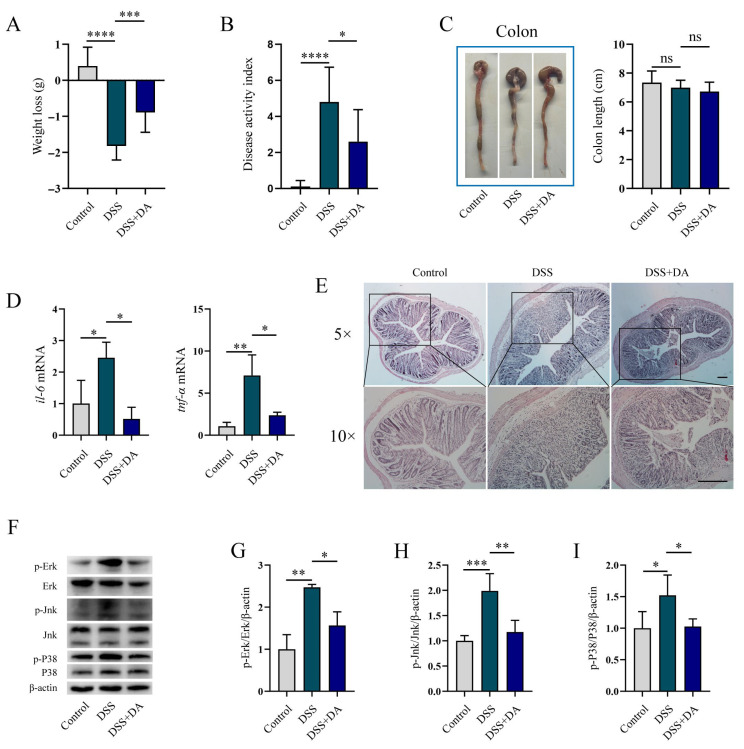
DA Mitigates Intestinal Inflammatory Injury in DSS-Induced Mice. (**A**–**C**) DA inhibits weight loss (**A**), reduces the disease activity index (**B**), and affects the colon length in DSS-induced WT mice (**C**) (*n* = 8). (**D**) qRT-PCR assay was used to analyze the mRNA levels of *il-6* and *tnf-α* in the colon tissue homogenate of DSS-induced WT mice. (**E**) H&E staining is employed to analyze the intestinal damage in the colon tissue of DSS-induced WT mice (*n* = 6). The 5× images are magnified 50 times, with a scale of 1000 μm. The 10× images are magnified 100 times, and the scale is 500 μm. (**F**–**I**) WB analysis is performed to detect the phosphorylation of Erk (**F**,**G**), Jnk (**F**,**H**), and P38 (**F**,**I**) in the colon tissue homogenate from DSS-induced WT mice. Data are presented as means ± SEM. * *p* < 0.05, ** *p* < 0.01, *** *p* < 0.001, **** *p* < 0.0001, and ns, not significant.

To assess whether DA alleviates intestinal inflammatory injury in vivo, a DSS-induced murine colitis model was utilized in this study. DSS Administration significantly decreased body weight and increased the DAI, which were mitigated by DA treatment (Figure 3A,B). Although DSS led to a tendency towards a reduction in colon length, this effect did not reach statistical significance (Figure 3C). Further analysis revealed that DA suppressed DSS-induced upregulation of *il-6* and *tnf-α* mRNA expression (Figure 3D). Histopathological examination (H&E staining) confirmed that DA significantly attenuated DSS-induced tissue damage (Figure 3E). DSS stimulation strongly enhanced the phosphorylation of Erk, Jnk, and P38, while DA supplementation significantly inhibited the phosphorylation of these kinases (Figure 3F–I).

### 3.4. DA Suppresses Oxidative Stress and Pyroptosis in DSS-Induced Mice

**Figure 4:**
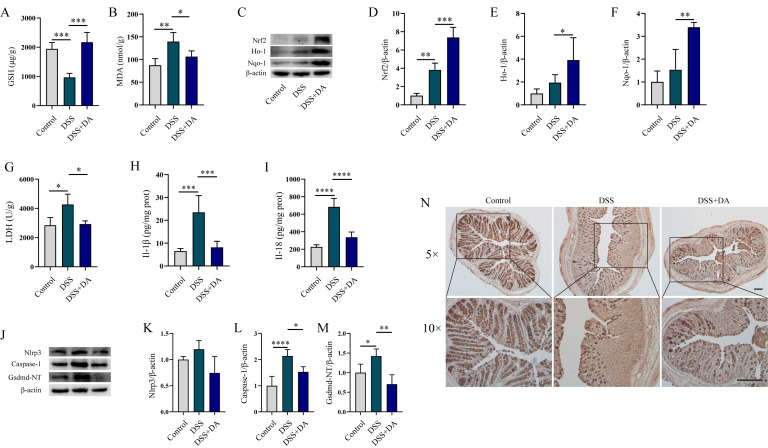
DA Suppresses Oxidative Stress and Pyroptosis in DSS-induced mice. (**A**,**B**) The contents of GSH and MDA were measured using GSH and MDA assay kit. (**C**–**F**) WB analysis was performed to detect the expression levels of Nrf2 (**C**,**D**), Ho-1 (**C**,**E**), and Nqo-1 (**C**,**F**) in DSS-induced WT mice. (**G**–**I**) The LDH assay kit (**G**), along with the Il-1β (**H**) and Il-18 (**I**) ELISA detection kits, were used to measure the levels of LDH, Il-1β, and Il-18 in colon tissue homogenates of DSS-induced WT mice. (**J**–**M**) WB analysis was performed to detect the expression of Nlrp3 (**J**,**K**), Caspase-1 (**J**,**L**), and Gsdmd-NT (**J**,**M**) in DSS-induced WT mice. (**N**) Immunohistochemical staining was used to detect the content of Muc2 protein in the colon tissues of DSS-induced WT mice (*n* = 6). The 5× images are magnified 50 times, with a scale of 1000 μm. The 10× images are magnified 100 times, and the scale is 500 μm. Data are presented as means ± SEM. * *p* < 0.05, ** *p* < 0.01, *** *p* < 0.001, and **** *p* < 0.0001.

Subsequently, we explored the impacts of DA on intestinal oxidative stress and pyroptosis in DSS-induced mice. DA significantly mitigated DSS-induced MDA elevation, increased GSH levels (Figure 4A,B). Furthermore, DA enhanced the expression of Nrf2, Ho-1, and Nqo-1 proteins (Figure 4C–F), suggesting its capacity to suppress DSS-induced intestinal oxidative stress. Considering that intestinal inflammatory damage is associated with pyroptosis, we examined whether DA could mitigate this process. DSS induction markedly increased the release of Il-1β, Il-18, and LDH (Figure 4G–I). In contrast, DA significantly decreased the secretion of these inflammatory mediators and LDH release. Further analysis revealed that DA significantly inhibited the DSS-induced upregulation of Nlrp3, Caspase-1, and Gsdmd-NT protein expression, demonstrating that DA suppresses DSS-induced pyroptosis (Figure 4J–M). Moreover, DA effectively increased the levels of Muc2 protein in the colon tissue of DSS-induced mice (Figure 4N).

### 3.5. Nrf2 Mitigates DSS-Induced Intestinal Inflammatory Injury

**Figure 5:**
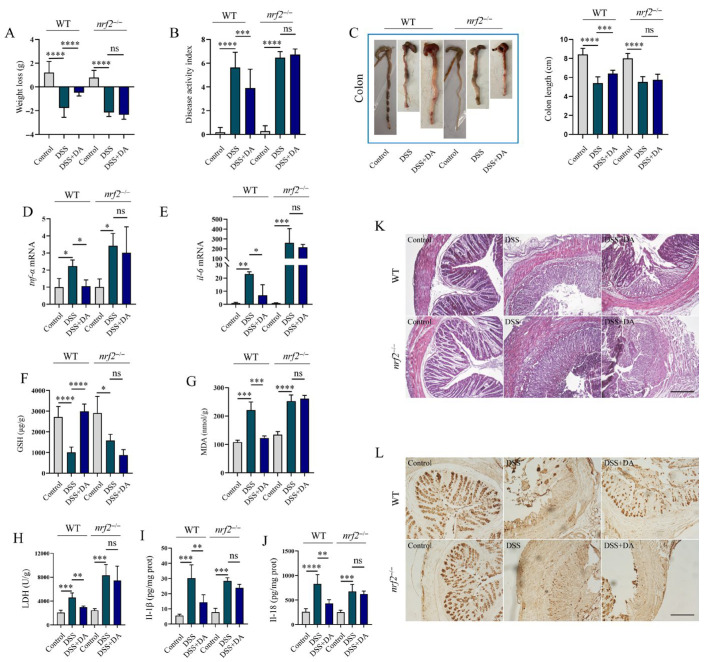
Nrf2 mitigates DSS-induced intestinal inflammatory injury. (**A**–**C**) DA inhibits weight loss (**A**), reduces the disease activity index (**B**), and affects the colon length in DSS-induced WT and *nrf2*^−/−^ mice (**C**) (*n* = 8). (**D**,**E**) qRT-PCR was performed to analyze the mRNA levels of *tnf-α* and *il-6* in the colon tissue homogenates of DSS-induced WT and *nrf2*^−/−^ mice. (**F**,**G**) The contents of GSH and MDA were measured using GSH and MDA assay kit. (**H**–**J**) The LDH assay kit, along with the Il-1β and Il-18 ELISA detection kits, were used to measure the levels of LDH, Il-1β, and Il-18 in the colon tissue homogenates from DSS-induced WT and *nrf2*^−/−^ mice. (**K**) H&E staining was used to analyze the intestinal damage in the colon tissues of DSS-induced WT mice (*n* = 6). (**L**) Immunohistochemical staining was used to detect the content of Muc2 protein in the colon tissues from DSS-induced WT mice (*n* = 6). Data are presented as means ± SEM. * *p* < 0.05, ** *p* < 0.01, *** *p* < 0.001, **** *p* < 0.0001, and ns, not significant.

**Figure 6:**
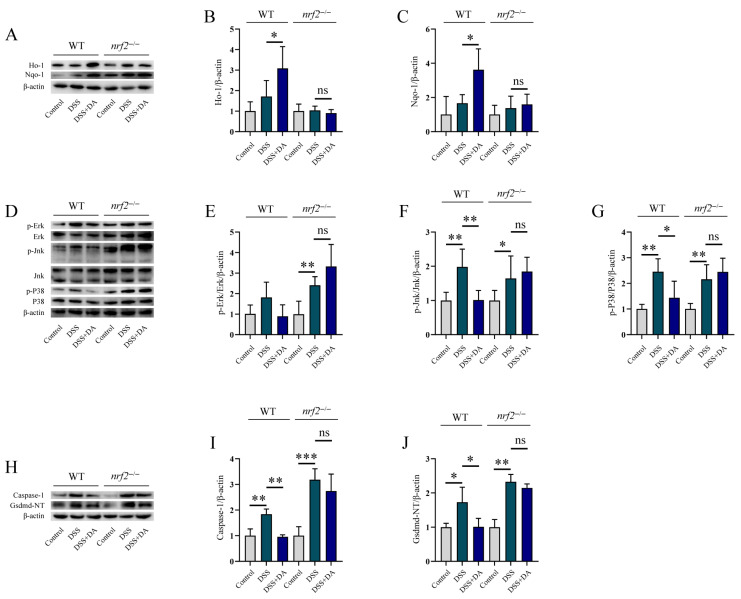
Nrf2 mitigates DSS-induced intestinal inflammatory injury. (**A**–**C**) WB analysis was performed to examine the effects of DA on the expression of Ho-1 (**A**,**B**), and Nqo-1 (**A**,**C**) The protein samples were derived from the homogenates of colon tissues from DSS-induced WT and *nrf2*^−/−^ mice. (**D**–**G**) WB was used to evaluate the effects of DA on the phosphorylation of Erk (**D**,**E**), Jnk (**D**,**F**), and P38 (**D**,**G**) signaling pathway. The protein samples were obtained from the homogenates of colon tissues from DSS-induced WT and *nrf2*^−/−^ mice. (**H**–**J**) WB analysis was used to assess the expression of Caspase-1 (**H**,**I**), and Gsdmd-NT (**H**,**J**). The protein samples were collected from the homogenates of colon tissues from DSS-induced WT and *nrf2*^−/−^ mice. Data are presented as means ± SEM. * *p* < 0.05, ** *p* < 0.01, *** *p* < 0.001, and ns, not significant.

Since Nrf2 is a common therapeutic target for alleviating oxidative stress and inflammation, we explored whether DA also exerts its effects through this pathway. Using a DSS-induced intestinal injury model in WT and *nrf2*^−/−^ mice, we found that DA significantly mitigated DSS-induced symptoms like weight loss, DAI increase, colon shortening, and histological damage in WT mice (Figure 5A–C,K) but not in *nrf2*^−/−^ mice. Further analysis indicated that in WT mice, DA inhibited the DSS-induced upregulation of *il-6* and *tnf-α* mRNA levels in colon tissue (Figure 5D,E). It also reduced MDA synthesis and LDH levels, while increasing the GSH level (Figure 5F–H). Moreover, the results showed that DA attenuated the release of Il-18 and Il-1β (Figure 5I,J), and prevented the DSS-induced reduction in colonic Muc2 protein levels (Figure 5L). Notably, all these beneficial effects of DA were not observed in *nrf2*^−/−^ mice. Additional research revealed that DA failed to induce the expression of the Nrf2-regulated antioxidant enzymes Ho-1 and Nqo-1 in the colon tissues of *nrf2*^−/−^ mice (Figure 6A–C). In *nrf2*^−/−^ mice, DA lost its ability to inhibit the phosphorylation of Erk, Jnk, and P38 (Figure 6D–G), which are components of the Mapk pathway, in response to DSS. Furthermore, DA was unable to inhibit the DSS-induced increase in the protein levels of the pyroptosis markers Nlrp3, caspase-1, and Gsdmd-NT in *nrf2*^−/−^ colon tissue (Figure 6H–J). Collectively, these findings demonstrate that DA alleviates DSS-induced intestinal injury, oxidative stress, inflammation, and pyroptosis predominantly in an Nrf2-dependent manner.

## 4. Discussion

IBD, predominantly including UC and CD [4,5], is a chronic and intractable inflammatory disorder of the gastrointestinal tract that affects millions of people globally [29]. Its pathogenesis involves a complex interplay among genetic predisposition, immune dysregulation, alterations in the gut microbiota, and environmental factors, all of which contribute to the disease susceptibility in both pediatric and adult populations [30]. Current therapeutic strategies for IBD primarily aim to suppress intestinal inflammation. These include Tnf-α antibodies [31], Il-12 and Il-23 antibodies [32], small molecule inhibitors of Janus kinase (Jak) and sphingosine-1-phosphate (S1p) receptor modulators [33], as well as the relatively conventional therapeutic drug 5-aminosalicylic acid [34]. Both antibody drugs and traditional drugs adhere to the treatment principle of inhibiting intestinal inflammation. For instance, they can inhibit the synthesis and secretion of Tnf-α or prevent its activation of Tnf receptors. Although there have been continuous advancements in IBD medications, bringing encouraging news to patients, IBD still remains one of the most difficult diseases to cure. Therefore, the search for safe and effective therapeutic drugs for IBD remains an urgent necessity.

In this study, we comprehensively assessed the therapeutic potential and underlying molecular mechanisms of DA in alleviating oxidative stress and inflammatory response. Using LPS-stimulated RAW264.7 macrophages as an in vitro model of intestinal injury, we demonstrated that DA significantly inhibited the production of pro-inflammatory cytokines *il-1β* and *il-6*. Given the hyper activation of the NF-κB p65 and Mapks (Erk, Jnk, and P38) signal pathway in both patients with IBD and DSS-induced murine models [35,36], we further examined the regulatory effects of DA on these signaling axes. DA effectively suppressed LPS-induced phosphorylation of Erk, Jnk, P38, and NF-κB p65, suggesting its anti-inflammatory effect might be mediated via the inhibition of the Mapks and NF-κB signaling pathways. Besides its anti-inflammatory properties, DA exhibited potent antioxidant activity. It significantly reduced the generation of ROS induced by LPS and downregulated the expression of iNos and Cox-2, which are key enzymes involved in the inflammatory response. Given the crucial role of the Nrf2 signaling pathway in cellular antioxidant defense [37], we explored the effect of DA on Nrf2 and its downstream effectors. DA strongly upregulated the expression of Nrf2, Ho-1, and Nqo-1, suggesting the activation of the Nrf2-mediated antioxidant defense system. To delve into the upstream regulatory mechanisms, we assessed the phosphorylation status of Ampk-α1 and Akt, both of which are recognized as promoting the stabilization and nuclear translocation of Nrf2. The phosphorylation of Akt enhances Nrf2 activity through dual mechanisms. First, it inhibits Gsk-3β, thereby preventing Nrf2 ubiquitination and proteasomal degradation [38]. Second, it directly facilitates the nuclear accumulation of Nrf2 and the subsequent transcription of antioxidant genes [39]. Similarly, Ampk phosphorylates Nrf2, promoting its nuclear translocation and transcriptional activity [40]. Notably, DA treatment significantly enhanced the phosphorylation of both Ampk-α1 and Akt, suggesting their involvement in DA-induced Nrf2 activation. Our findings indeed indicated that treatment with DA facilitates the nuclear translocation of Nrf2. Collectively, our findings demonstrate that DA exerts protective effects against oxidative stress and inflammation primarily by activating the Nrf2 pathway via Ampk/Akt signaling, while concurrently suppressing the Mapks and NF-κB pathways.

To further assess the protective effects of DA against intestinal oxidative stress and inflammation, we developed a murine colitis model using DSS to mimic IBD. DA Administration markedly ameliorated key pathological characteristics of intestinal injury. Specifically, it alleviated weight loss, reduced the DAI, and mitigated intestinal inflammatory damage. Mechanistically, DSS treatment activated the Nrf2/Ho-1/Nqo-1 antioxidant pathway to some extent, and DA further enhances its activation. Correspondingly, DA significantly decreased DSS-induced MDA accumulation, which is a marker of lipid peroxidation and oxidative damage. Meanwhile, DA elevated GSH levels, indicating that it enhanced cellular antioxidant capacity. MDA and GSH are crucial indicators of redox homeostasis, their imbalance mean the occurrence of oxidative stress [41]. Furthermore, DSS strongly promoted the phosphorylation of Erk, Jnk, and P38 Mapks, which are crucial signaling pathway. Conversely, pretreatment with DA effectively suppressed their activation. Notably, DA also decreased the DSS-induced expression of the Nlrp3 inflammasome, Caspase-1, and Gsdmd-NT, resulting in a reduction in the maturation and release of Il-1β and Il-18. These results indicated that DA inhibits Nlrp3-dependent pyroptosis, a lytic form of inflammatory cell death associated with the pathogenesis of IBD. Previous research has demonstrated that suppression Nlrp3-mediated pyroptosis can alleviate intestinal inflammation and epithelial barrier disruption [42,43]. Overall, our findings indicate that DA ameliorates DSS-induced colitis by reducing oxidative stress, dampening Mapk-driven inflammatory signaling pathways, and inhibiting Nlrp3 inflammasome activation and subsequent pyroptosis. Although these data underscore the multi-target therapeutic potential of DA, the exact molecular targets through which it exerts these protective effects remain to be fully elucidated.

Excessive production of ROS activates the Mapks signaling pathway, triggering inflammatory response [44]. Nrf2 serves as a key regulator of cellular antioxidant defenses and has emerged as a promising therapeutic target. Activation of Nrf2 not only mitigates oxidative damage but also exerts potent anti-inflammatory effects. For instance, ursolic acid has been shown to bind to Kelch-like ECH-associated protein 1 (Keap1), facilitating its dissociation from Nrf2 and enabling Nrf2 to translocate into the nucleus, thereby reducing myocardial ischemia–reperfusion injury [45]. Similarly, apremilast ameliorates trinitrobenzene sulfonic acid (TNBS)-induced colitis in mice by activating the Nrf2 signaling pathway in intestinal glial cells [46]. To explore the mechanistic role of Nrf2 in the protective effects of DA against intestinal inflammation, we established a DSS-induced colitis model using *nrf2*^−/−^ mice. Our findings indicated that genetic ablation of Nrf2 completely eliminates the protective effects of DA. In the absence of Nrf2, DA failed to inhibit the excessive generation of MDA, elevate the level of GSH, or promote the expression of Ho-1 and Nqo-1. Moreover, DA no longer exerts its beneficial effects on intestinal inflammatory injury. Specifically, it cannot alleviate weight loss and colon shortening, nor can it decrease the DAI and histological score. Mechanistically, the lack of Nrf2 negates DA’s inhibitory effects on both the Mapks and Nlrp3 inflammasome signaling pathways. Specifically, in *nrf2*^−/−^ mice, DA no longer suppresses the phosphorylation of Erk, Jnk, and P38. Additionally, its capacity to inhibit Nlrp3-dependent pyroptosis is lost, emphasizing the critical dependence of this function on Nrf2. Oxidative stress is a well-recognized activator of Nlrp3 inflammasome-mediated pyroptosis via multiple interconnected mechanisms. At the mitochondrial level, excessive production of ROS disrupts mitochondrial homeostasis. This leads to Nlrp3 conformational activation and oligomerization, a prerequisite for inflammasome assembly and Caspase-1 activation [47]. Additionally, thioredoxin-interacting protein (Txnip), which is upregulated under oxidative stress conditions, directly interacts with Nlrp3 to facilitate inflammasome formation [48]. ROS also act as a second messengers in the Mapk signaling cascade. ROS facilitates the dimerization and auto-phosphorylation of receptor tyrosine kinases (Rtks), leading to Ras activation. Subsequently, Raf (Mapkkk), Mek (Mapkk) are phosphorylated in sequence, and ultimately Erk, Jnk, or P38 are phosphorylated [49].

While the majority of studies on DA have focused on its pharmacodynamics, specific signaling pathways, or individual targets, a comprehensive understanding of their interrelationships is still lacking. Future research should incorporate network pharmacology and multi-omics technologies to systematically map the “component-target-disease” interaction network, facilitating a thorough analysis of DA’s mechanisms [17]. Clinical trials reveal that dehydroandrographolide succinate (DAS), a derivative of DA, exhibits rapid distribution, swift elimination, nonlinear pharmacokinetics, and favorable tolerability [50,51]. Despite its promising anti-inflammatory effects and safety profile, DAS encounters challenges such as a short half-life and low urinary excretion of the parent compound, suggesting complex *in vivo* metabolism. Exploring methods to extend its half-life or further investigating the functions of its *in vivo* metabolites may represent the direction for future research endeavors.

## 5. Conclusions

*In vivo* experiments revealed that DA effectively alleviated LPS-induced oxidative stress and inflammatory responses. Additionally, DA was found to inhibit DSS-induced intestinal oxidative stress, inflammation, and pyroptosis. Notably, these protective effects were completely eliminated in Nrf2-deficient mice, suggesting a crucial dependence on the Nrf2 signaling pathway. These findings prompt us to hypothesize that DA mainly exerts its antioxidant and anti-inflammatory effects by activating Ampk-α1/Akt signaling pathway. This activation subsequently promotes the activation of the Nrf2 signaling pathway, thereby suppressing the accumulation of ROS. Then, the alleviated oxidative stress mitigates the abnormal activation of the Mapks signaling pathway and inhibits the assembly and activation of the Nlrp3 inflammasome. Ultimately, this prevents pyroptosis and alleviates intestinal inflammatory damage.

## Data Availability

The original contributions presented in this study are included in the article/Supplementary Materials. Further inquiries can be directed to the corresponding authors.

## Supplementary Materials

The following supporting information can be downloaded at: https://www.mdpi.com/article/10.3390/biom15111580/s1, Original Western blot images.

## Abbreviations

The following abbreviations are used in this manuscript:

DA	Dehydroandrographolide
LPS	Lipopolysaccharide
*tnf**-α*	*Tumor Necrosis Factor-α*
*il-6*	*Interleukin**-6*
*il-1β*	*I**nterleukin-**1**β*
Erk	Extracellular Signal-Regulated Kinase
Jnk	C-Jun N-Terminal Kinase
P38	p38 Mitogen-Activated Pro-Tein Kinase
NF-κB	Nuclear Factor kappa-light-chain-enhancer of activated B cells
Cox-2	Cyclooxygenase-2
iNos	Inducible Nitric Oxide Synthase
Nrf2	Nuclear Factor Erythroid 2-Related Factor 2
Nqo-1	Nad(P)H Quinone Dehydrogenase 1
Ho-1	Heme Oxygenase-1
Akt	Protein Kinase B
Ampk-α1	5′-Adenosine Monophos-Phate-Activated Protein Kinase-α1
WT	Wild type
DAI	Disease Activity Index
Nlrp3	Nod-Like Receptor Family Pyrin Domain-Containing 3
*nrf2*^−/−^	*nrf2* Knockout
IBD	Inflammatory Bowel Disease
UC	Ulcerative Colitis
CD	Crohn’s Disease
DMSO	Dimethyl Sulfoxide
RIPA	Radioimmunoprecipitation Assay
PMSF	Phenylmethylsulfonyl Fluoride
DMEM	Dulbecco’s Modified Eagle’s Medium
FBS	Fetal Bovine Serum
H&E	Hematoxylin And Eosin
ECL	Enhanced Chemiluminescence
ROS	Reactive Oxygen Species
DCFH-DA	2′,7′-Dichlorodihydrofluorescein Diacetate
MDA	Malondialdehyde
GSH	Glutathione
LDH	Lactate Dehydrogenase
SEM	Standard Error Of The Mean
ANOVA	One-Way Analysis Of Variance
LSD	Least Significant Difference
Mapk	Mitogen-Activated Protein Kinase
Jak	Janus Kinase
S1p	Sphingosine-1-Phosphate
TNBS	Trinitrobenzene Sulfonic Acid
Txnip	Thioredoxin-Interacting Protein
Rtks	Receptor Tyrosine Kinases
