# Supplementary figures and images for: Dehydroandrographolide Alleviates Oxidative Stress, Inflammatory Response, and Pyroptosis in DSS-Induced Colitis Mice by Modulating Nrf2 Signaling Pathway
Source: Biomolecules. 2025 Nov 10;15(11):1580. doi: 10.3390/biom15111580 (PMC12650035; doi:10.3390/biom15111580)

### Figure S1

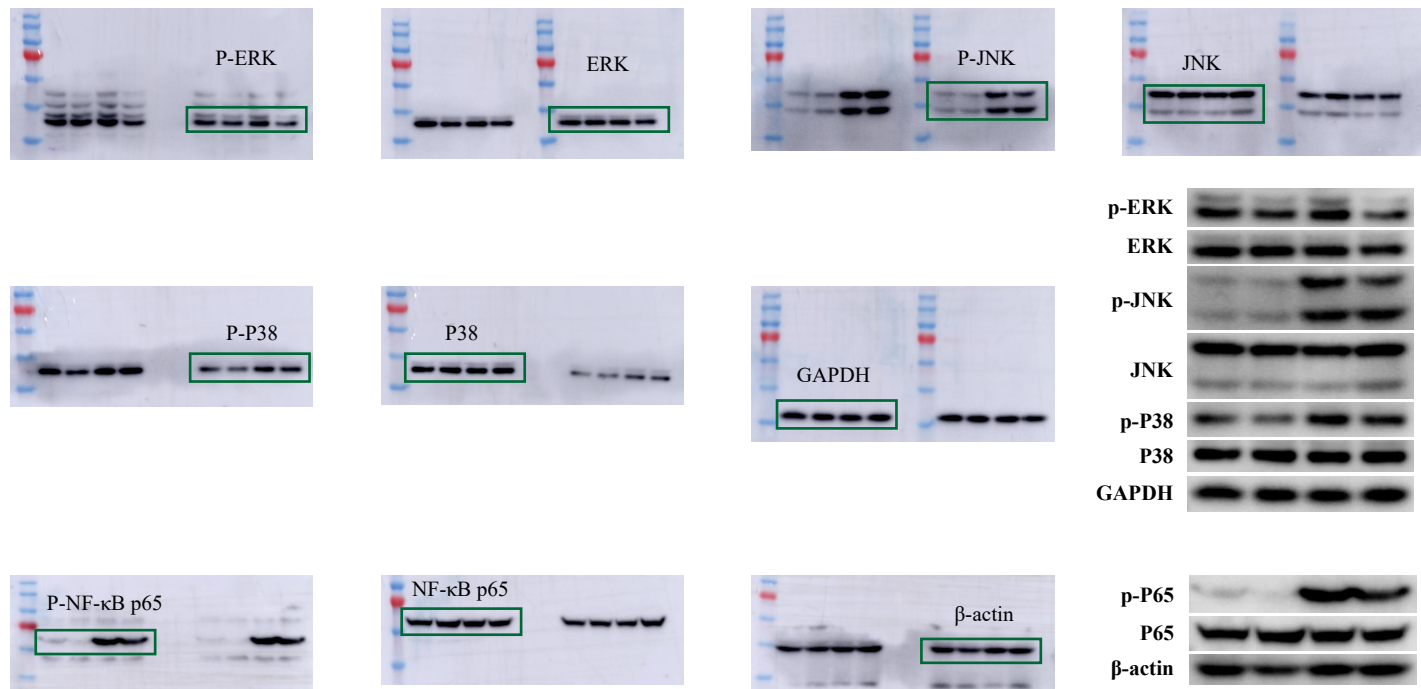

**Figure S2**

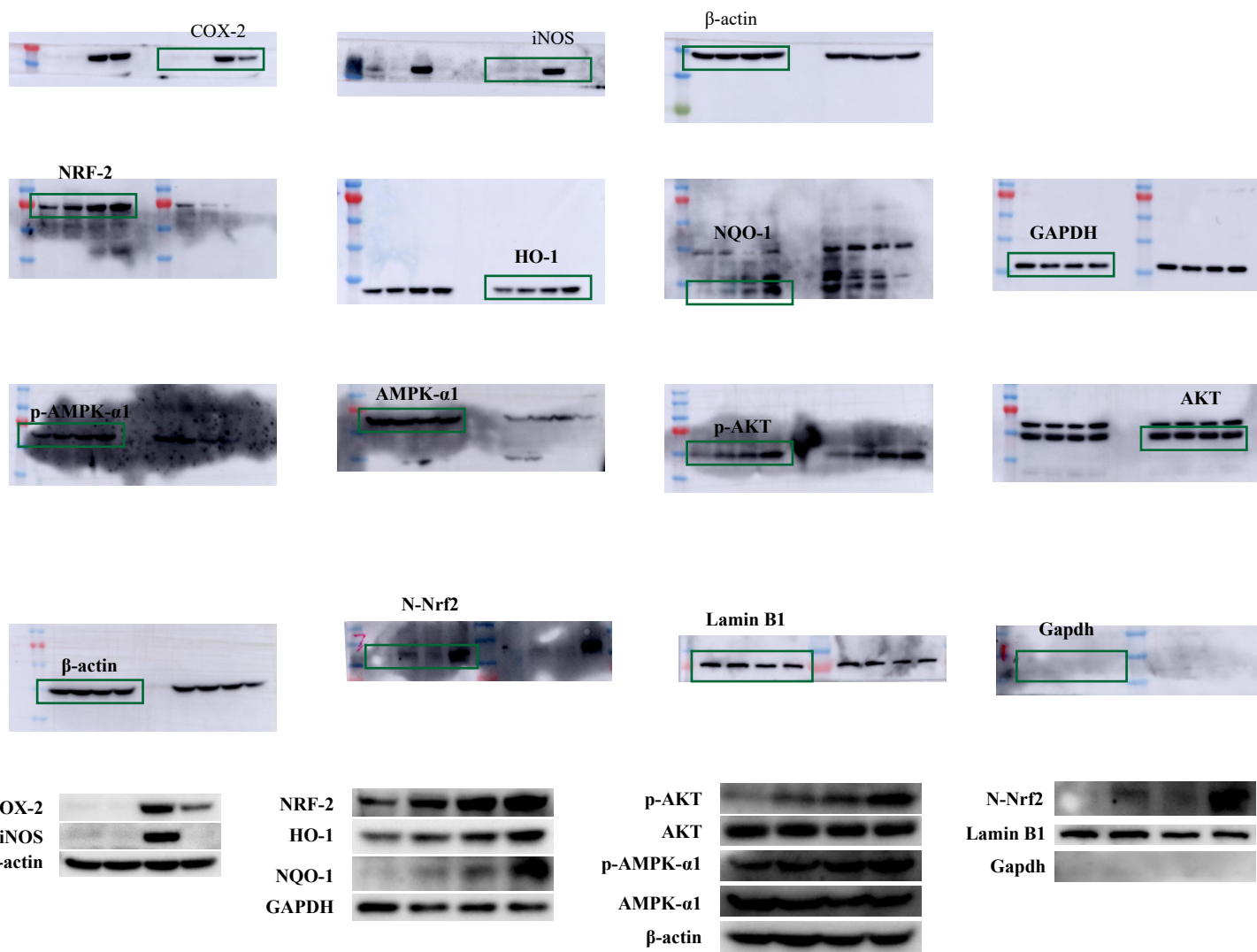

Figure S3

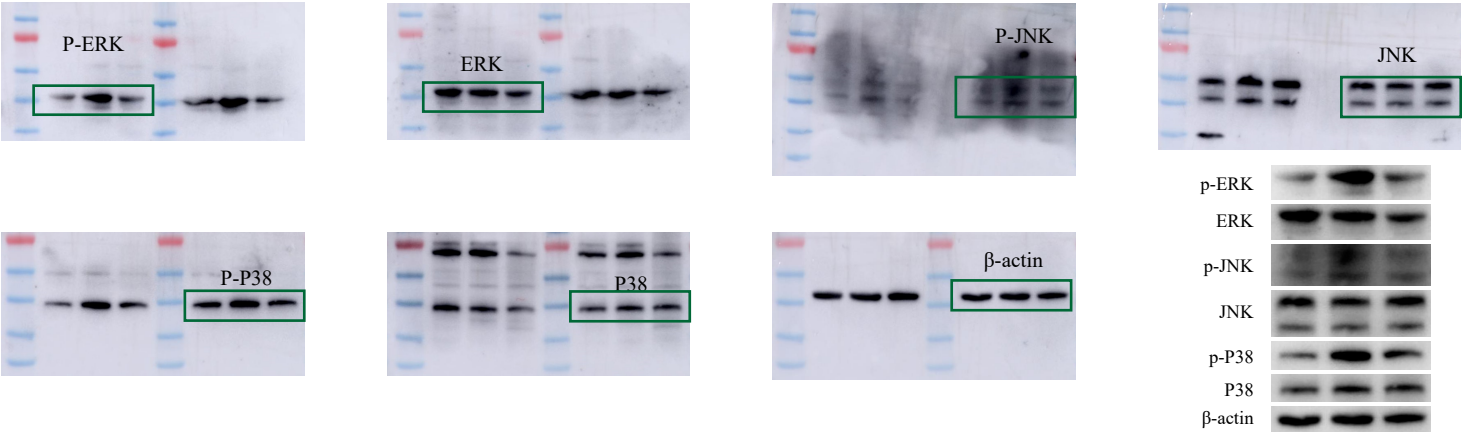

Figure S4

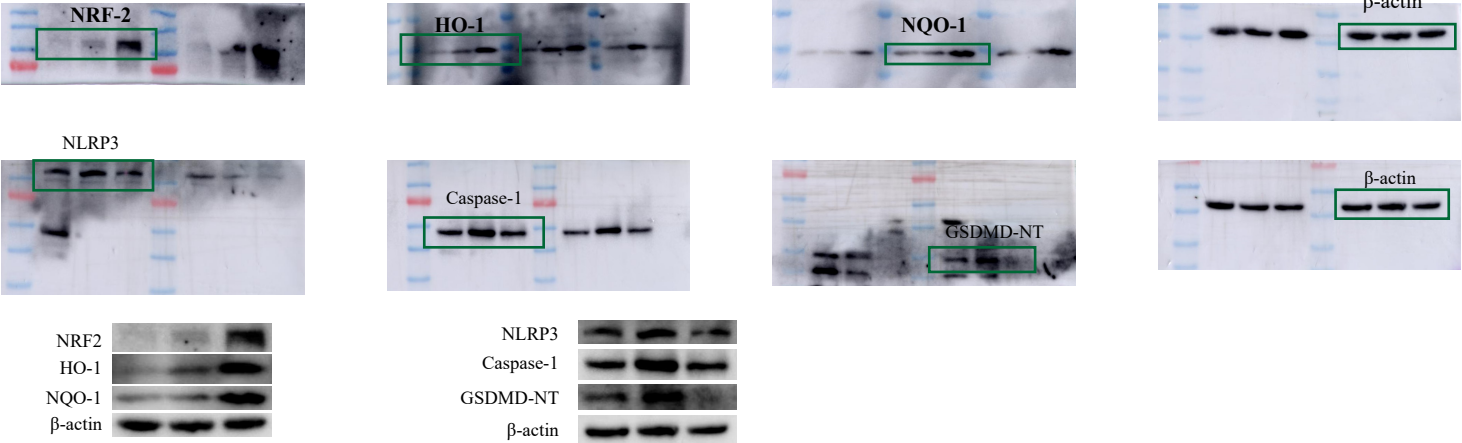

Figure S6

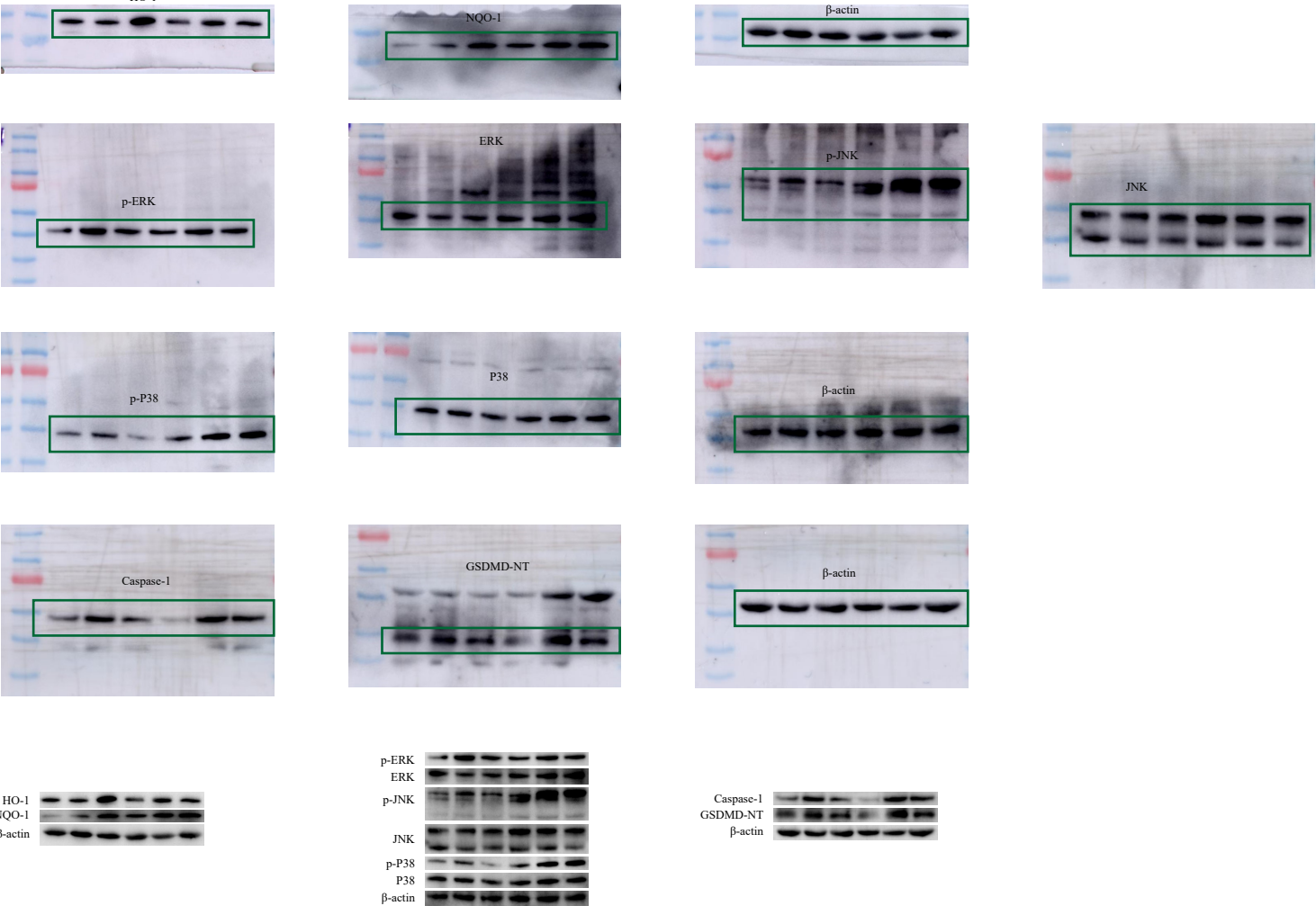

Supplement: Supplementary file 1 [file biomolecules-15-01580-s001.zip › Original Western blot images.pdf]
